# Evaluation of fungal degradation of wheat straw cell wall using different analytical methods from ruminant nutrition perspective

**DOI:** 10.1002/jsfa.9634

**Published:** 2019-03-13

**Authors:** Nazri Nayan, Gijs van Erven, Mirjam A Kabel, Anton SM Sonnenberg, Wouter H Hendriks, John W Cone

**Affiliations:** ^1^ Animal Nutrition Group Wageningen University & Research Wageningen The Netherlands; ^2^ Laboratory of Food Chemistry Wageningen University & Research Wageningen The Netherlands; ^3^ Plant Breeding Wageningen University & Research Wageningen The Netherlands

**Keywords:** white‐rot fungi, lignin, carbohydrates, lignin quantification, *in vitro* gas production, pyrolysis‐GC/MS

## Abstract

**BACKGROUND:**

White rot fungi have been used to improve the nutritive value of lignocellulose for ruminants. In feed analysis, the Van Soest method is widely used to determine the cell wall contents. To assess the reliability of this method (Method A) for determination of cell wall contents in fungal‐treated wheat straw, we compared a combined monosaccharide analysis and pyrolysis coupled to gas chromatography with mass spectrometry (Py‐GC/MS) (Method B). Ruminal digestibility, measured as *in vitro* gas production (IVGP), was subsequently used to examine which method explains best the effect of fungal pretreatment on the digestibility of wheat straw.

**RESULTS:**

Both methods differed considerably in the mass recoveries of the individual cell wall components, which changed on how we assess their degradation characteristics. For example, Method B gave a higher degradation of lignin (61.9%), as compared to Method A (33.2%). Method A, however, showed a better correlation of IVGP with the ratio of lignin to total structural carbohydrates, as compared to Method B (Pearson's *r* of −0.84 *versus* −0.69). Nevertheless, Method B provides a more accurate quantification of lignin, reflecting its actual modification and degradation. With the information on the lignin structural features, Method B presents a substantial advantage in understanding the underlying mechanisms of lignin breakdown. Both methods, however, could not accurately quantify the cellulose contents – among others, due to interference of fungal biomass.

**CONCLUSION:**

Method A only accounts for the recalcitrant residue and therefore is more suitable for evaluating ruminal digestibility. Method B allows a more accurate quantification of cell wall, required to understand and better explains the actual modification of the cell wall. The suitability of both methods, therefore, depends on their intended purposes. © 2019 The Authors. *Journal of The Science of Food and Agriculture* published by John Wiley & Sons Ltd on behalf of Society of Chemical Industry.

## INTRODUCTION

Unlocking the lignin–carbohydrate complex in the cell wall is a key step in improving the utilization of agricultural biomass for various applications, including animal feed.^1^, ^2^, ^3^ Due to an increased demand for eco‐friendly approaches, biological methods, including the use of white‐rot fungi have received much attention in recent years.^4^, ^5^ Many studies reported considerable successes in using fungi such as *Ceriporiopsis subvermispora*, *Lentinula edodes* and *Pleurotus eryngii* for improving the ruminal digestibility of biomasses.^4^, ^6^, ^7^ These studies mainly rely on the classical Van Soest *et al.* 8 method to assess the capability of a particular fungus in modifying the cell wall contents – hence, explaining the subsequent ruminal digestibility. Since there is a growing interest for future research on the application of fungi, it is important to ascertain the reliability of this commonly used method. Using wheat straw as a model substrate, this article deals with the relevance of different analytical methods in understanding the degradation of cell wall by white‐rot fungi.

While the Van Soest method is practical and provides a good estimate of the cell wall contents, it is, however, unspecific. For instance, acid‐detergent lignin (ADL) – a fraction of the Van Soest method, only represents the recalcitrant lignin content in the residue, and does not quantify the acid soluble lignin.^9^ We hypothesize that during the fungal growth, parts of the remaining carbohydrate and lignin become more soluble and are subsequently removed in the (acid‐) soluble fractions, thus further interfere with the quantification of different cell wall components from the Van Soest method. In addition, parts of the fungal biomass components that have structural resemblance to that of substrate may further complicate the outcomes. To investigate the relevance of the Van Soest method, we performed specific analyses for both lignin and structural carbohydrates. Pyrolysis coupled to gas chromatography with mass spectrometry (Py‐GC/MS) has been shown to be a powerful tool in estimating the lignin content, while simultaneously providing information on the lignin structural features.^5^, ^10^ Recent development of a highly accurate Py‐GC/MS technique, which uses carbon‐13 (^13^C) lignin as internal standard, allows an accurate quantification of the lignin content.^11^ This precise technique has also been used to study the mechanisms of different fungal species in modifying lignin structure.^12^ Meanwhile, cellulose and hemicellulose can be also quantified using constituent monosaccharides analysis.^13^

From ruminant nutrition perspective, the digestibility of the fungal‐treated wheat straw is an important indicator and hence, was used as the basis of comparison between different analytical methods in this study. The *in vitro* gas production (IVGP) is a robust and effective technique to assess *in vitro* digestibility of substrates in the rumen.^14^ It has been widely used in feed evaluation studies, including assessment of fungal‐treated agricultural biomasses.^7^, ^15^ Recently, we have screened for high potential strains of *C. subvermispora*, *P. eryngii* and *L. edodes*, based on their abilities to improve IVGP of wheat straw.^16^ These high potential strains showed a considerable variation in degrading the cell wall contents, particularly the ADL content of wheat straw (22.3–52.2%), providing a good range of data for the purpose of this study.

The aim of this study was to evaluate the relevance of different analytical methods in assessing fungal degradation of wheat straw cell wall with respect to mass recoveries and relationship with the IVGP. We also presented the advantages and disadvantages of using both methods and their practical applications when assessing fungal pretreatment of wheat straw. Hereto, two methods were compared: (i) Method A, based on Van Soest *et al.* 8; (ii) Method B, by combined methods of assessing lignin and carbohydrates, based on Py‐GC/MS 11, 17 and constituent monosaccharide analysis,^13^ respectively.

## MATERIALS AND METHODS

### Preparation of the fungal‐treated wheat straw

Two high potential strains from three different fungal species were previously selected based on their ability to improve IVGP of wheat straw: CS1 (CBS 347.63) and CS12 (ME‐485) strains of *C. subvermispora*, PE3 (Mycelia2600) and PE6 (AL04) of *P. eryngii* and LE8 (sh 03/08) and LE10 (LE75) of *L. edodes*.^16^ Procedures for the fungal strain preparations and treatment of the wheat straw have been described previously in detail.^4^ All strains were maintained on malt extract agar before a piece of that agar (1.5 cm × 2.0 cm) was used to prepare the spawn for each fungus. The spawn was prepared using sterilized sorghum grains and was incubated at 24 °C for 5 weeks. Organic wheat straw was purchased from a local farmer in the Netherlands, and chopped into approximately 3 cm pieces. The wheat straw was soaked in water for 3 days at room temperature and excess water was drained for 5 h. Adjustments were made based on the final moisture content of the straw (∼74% *w*/*w*), for each container (Combiness, Nevele, Belgium) to contain 90.2 ± 0.3 g of dry matter (DM). After autoclaving at 121 °C for 1 h, the straw was inoculated with the prepared spawn at 10% of the dry weight. The wheat straw (treated and untreated with fungi) was incubated in triplicate aerobically at 24 °C for 7 weeks in a climate‐controlled chamber. All samples were freeze‐dried and ground over a 1 mm sieve using a cross beater mill (100AN, Peppink, Olst, The Netherlands).

### In vitro gas production (IVGP)

The IVGP – expressed as milliliters per gram of organic matter (OM), was performed according to Cone *et al.* 14 In brief, ∼0.5 ± 0.01 g of sample was incubated in 60 mL buffered rumen fluid at 39 °C. The incubation was carried out for 72 h and the gas production was recorded automatically.

### Conventional feed analyses: ‘Van Soest’

Samples were dried in an oven at 103 °C to determine the DM content (ISO 6496, 1999); and then further combusted at 550 °C for 3 h in a muffle furnace for the ash content (ISO 5984, 2002). Nitrogen content was determined by the Kjeldahl method (ISO 5983, 2005) and crude protein was calculated as N × 6.25. Compositional analysis of the fiber contents was carried out according to Van Soest *et al.* 8, in an ANKOM 200 fiber analyzer (ANKOM Technology, New York, USA). Neutral‐detergent fiber (NDF) was determined using a heat‐stable amylase (thermamyl) and alcalase. Acid‐detergent fiber (ADF) and ADL were determined by boiling the sample in an acid‐detergent solution, and to determine ADL the residue was further treated with 72% *v*/*v* sulfuric acid (H_2_SO_4_). All fiber contents were corrected for ash. Hemicellulose was calculated by subtracting ADF from NDF values, while cellulose was calculated as the difference between ADF and ADL. The cell wall contents were expressed on dry matter basis (% *w*/*w* DM). Absolute amounts were calculated from the remaining freeze‐dried materials (in grams), which were corrected for their DM contents. The Van Soest method resulted in variations of the ADL content (<10%) among different biological replicates of the same treatment, while cellulose and hemicellulose showed less than 5% variation. Therefore, prior to the monosaccharides and Py‐GC/MS analyses, an equal amount of each biological replicate belonging to the same treatment was thoroughly mixed into one sample, allowing more accurate lignin and carbohydrates analyses.

### Carbohydrate content and composition: ‘Englyst’

The carbohydrate content and composition was determined in duplicate according to Englyst and Cummings,^13^ using inositol as an internal standard. Samples were treated with 72% (*w*/*w*) H_2_SO_4_ (1 h, 30 °C) followed by hydrolysis with 1 mol L^−1^ H_2_SO_4_ for 3 h at 100 °C. The constituent sugars released were analyzed as their alditol‐acetates using gas chromatography (ThermoScientific, Waltham, MA, USA) and determined as anhydrocarbohydrates. A standard with a known concentration of glucose, galactose, mannose, arabinose, rhamnose and xylose was taken along in the procedure. The uronic acids released after the acid hydrolysis step, were determined in duplicate as anhydrouronic acid by an automated meta‐hydroxydiphenyl assay^18^ with addition of sodium tetraborate using an auto‐analyzer (Skalar Analytical BV, Breda, The Netherlands). Glucuronic acid (Fluka AG, Buchs, Switzerland) was used as a reference (0–100 µg mL^−1^). Glucan was considered as a total cellulosic polymer, while glucuronoarabinoxylan (GAX) as the hemicellulosic polymer by summing up xylan, arabinan and uronic acid.

### Semi‐quantitative and quantitative pyrolysis

Prior to Py‐GC/MS, ground wheat straw (1 mm) was ball‐milled in a MM200 mixer mill (Retsch, Haan, Germany). Detailed procedures have been described previously.^11^, ^17^ Pyrolysis was performed with an EGA/PY‐3030D Multi‐shot pyrolyzer (Frontier Laboratories, New Ulm, MN, USA) equipped with an AS‐1020E Autoshot auto‐sampler. The pyrolyzer was coupled to a GC/MS, using a Trace GC, coupled to a DSQ‐II mass spectrometer (both Thermo Scientific, Waltham, MA, USA). Pyrolysis of samples (∼80 µg) was performed at 500 °C for 1 min. Pyrolysis products were injected into the column via a split/splitless injection (at 250 °C) with a splitratio of 1:133 and helium was used as carrier gas. The GC oven was programmed at 270 °C for 15 min. MS detection was used with a scan range of 50–550 *m/z* and a scan rate of 4.0 scans s^−1^. Compounds were identified by comparing the retention time and mass spectra with standards, the NIST library and published data.^19^ For semi‐quantitative analysis (samples of weeks 0, 1, 3 and 7), pyrograms were processed with Xcalibur 2.2 software. Areas were normalized by dividing by corresponding relative response factors (RRFs), multiplied with the molecular weight of the respective compound and summed.^11^ Lignin content was estimated on the basis of the total area of lignin‐derived pyrolysis products and compared to a wheat straw reference sample with known total Klason lignin content (20.5% *w*/*w*).^17^

Only samples of weeks 0 and 7 were subjected to the quantitative Py‐GC/MS, using the previously described procedures.^11^ Briefly, 10 µL of a ^13^C lignin internal standard (IS) solution (1 mg mL^−1^) was mixed with ∼80 µg of sample and dried before analysis. Lignin‐derived pyrolysis products were monitored in the selected ion monitoring (SIM) mode on the two most abundant fragments per compound (both ^12^C and ^13^C). The area for each compound was normalized by dividing by the RRF. The RRF values were updated to system performance by recalculation to obtain an identical relative abundance of lignin‐derived pyrolysis products of the ^13^C IS added to the wheat straw reference sample. Lignin content (% *w*/*w*) was determined as the sum of lignin‐derived pyrolysis products by the established formula.^11^ Relative abundances of lignin‐derived pyrolysis products were based on areas normalized for the ^13^C analogues from the IS present in the same sample to distinguish matrix and treatment effects. Areas were not corrected for molecular weight. Compounds were classified according to their structural features (see Supporting Information Table S1) and summed. All samples were prepared and analyzed in triplicate. A complete characterization of lignin structural moieties (e.g. unsubstituted and C_α_‐oxidized compounds) has also been published in Van Erven et al.^12^

### Statistics

Data were analyzed using the general linear model (analysis of variance, ANOVA) in SAS 9.3, followed by *post hoc* multiple comparison with Fisher's least significance differences. The statistical model used was as follows:

$$Y_{\mathrm{ijk}}=µ+{\mathrm{SP}}_{i}+{\mathrm{ST}}_{j\left(i\right)}+\tau_{k\left(i\;j\right)}+\epsilon_{\mathrm{ijk}}$$

where *Y*_*ijk*_ represents the response variable *ijk*; *µ*, overall mean; *SP*_*i*_, the effect of species *i*; *ST*_*j(i)*_, the effect of strain *j* nested within species *i*; *τ*_*k(i j)*_, effect of week *k*; *ϵ*_*ijk*_, residual error with a mean of zero and variance *σ* ^2^. Thus, *SP*_*i*_ was considered a fixed effect, *ST*_*j(i)*_ and *τ*_*k(i j)*_ as random effects. Pearson Product–Moment Correlation (*r*) coefficients were also determined among the measured variables. Probability values below 5% were considered significant. No ANOVA was applied on the analytical replicates data of the mixed samples (monosaccharides and Py‐GC/MS analyses). However, the coefficient of variation (CV) was calculated as a measure of proportional error.^20^

## RESULTS AND DISCUSSION

**Figure 1:**
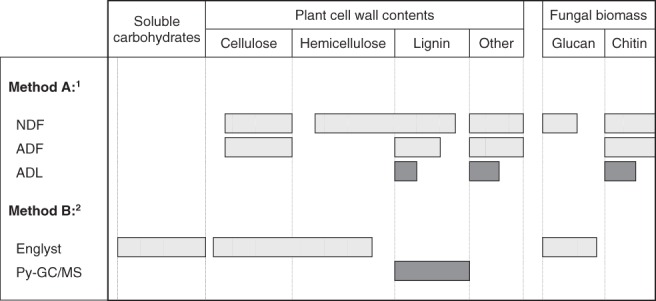
Schematic representation indicating mass recoveries of different cell wall components, measured using two different methods: (i) Method A based on Van Soest *et al.*;^8^ cellulose (ADF–ADL), hemicellulose (NDF–ADF); and (ii) Method B using the Englyst and Cummings^13^ method for carbohydrate analysis and Py‐GC/MS for lignin.^11^ NDF, neutral‐detergent fiber; ADF, acid‐detergent fiber; ADL, acid‐detergent lignin. Other plant cell wall contents include pectic polysaccharides and resistant starch. Glucan (*α*‐ or *β*‐) and chitin are components of pure fungal biomass. Length of the bars indicate expected amount of cell wall components that are recovered by each method.

The cell wall contents of wheat straw, treated with six high potential fungal strains for 7 weeks were assessed using two methods: (i) Method A was based on Van Soest *et al.* 8; and (ii) Method B represents two specific analyses for lignin and carbohydrates, using ^13^C quantitative Py‐GC/MS (and semi‐quantitative for weekly data)^11^, ^17^ and the Englyst method,^13^ respectively. Figure 1 illustrates different components (belonging to substrate and fungi) that would be recovered in both methods. Here, we assessed the two methods by comparing the cell wall contents and their degradation (in absolute amounts); and later discussing their differences in view of ruminal digestibility of the fungal‐treated wheat straw.

### Analysis of the cell wall using the two methods

The cell wall contents of wheat straw, with and without fungal pretreatment after 7 weeks are provided in Table 1. For the untreated wheat straw (control), the cell wall contents were in the range of previously reported values (on a DM basis, *w*/*w*) for cellulose (46–50%), hemicellulose (26–31%) and ADL (7–9%) (Method A).^4^, ^7^, ^15^ Similarly, the cell wall contents determined in Method B were comparable to previous reports on cellulose (34–43%) and hemicellulose (26–35%).^21^, ^22^, ^23^ The lignin contents, determined by ^13^C quantitative Py‐GC/MS, were similar to the total Klason lignin content of wheat straw (∼25%).^10^, ^17^ These comparable results demonstrate reliability of the cell wall analyses performed on the bioprocessed wheat straw in the present study.

**Table 1:** The in vitro gas production (IVGP) and chemical composition (mean ± SD), as determined using two methods, for wheat straw treated with different fungal strains for 7 weeks

		*Ceriporiopsis subvermispora* strain		*Pleurotus eryngii* strain		*Lentinula edodes* strain		
Parameters	Control	1	12	3	6	8	10	CV
IVGP (mL g^−1^ OM)	227.7^a^ ± 11.58	313.2^e^ ± 6.56	284.2^cd^ ± 16.07	252.4^b^ ± 5.47	263.0^bc^ ± 5.90	297.7^de^ ± 10.23	287.3^d^ ± 15.62	5.72
DM (%)	21.4^b^ ± 0.05	20.0^a^ ± 0.01	20.9^b^ ± 0.23	21.0^b^ ± 0.18	21.4^b^ ± 0.11	20.0^a^ ± 0.29	20.2^a^ ± 0.64	1.65
Ash (% *w*/*w* DM)	3.3^a^ ± 0.04	4.3^e^ ± 0.12	4.1^cd^ ± 0.07	4.1^de^ ± 0.08	4.0^bc^ ± 0.10	3.9^b^ ± 0.07	4.0^bc^ ± 0.06	2.10
Compositions, % *w*/*w* DM (absolute amount, g)								
CP	1.6^a^ ± 0.03	2.2^a^ ± 0.08	2.0^a^ ± 0.02	2.2^a^ ± 0.09	2.1^a^ ± 0.10	2.2^a^ ± 0.10	2.2^a^ ± 0.02	25.54
	(1.5)	(1.9)	(1.8)	(1.9)	(1.8)	(1.8)	(1.9)	
Method A								
Cell	50.0^ab^ ± 0.44	50.2^ab^ ± 0.10	49.7^a^ ± 0.92	50.8^b^ ± 0.96	52.4^c^ ± 0.36	53.6^d^ ± 0.10	52.7^c^ ± 0.14	1.05
	(45.1)	(42.3)	(44.4)	(44.3)	(46.1)	(44.9)	(44.5)	
Hcell	29.1^e^ ± 0.92	15.6^a^ ± 0.66	20.2^c^ ± 0.96	23.3^d^ ± 0.20	23.9^d^ ± 0.43	18.2^b^ ± 0.86	20.3^c^ ± 0.35	3.08
	(26.3)	(13.2)	(18.1)	(20.3)	(21.0)	(15.2)	(17.1)	
ADL	8.5^d^ ± 0.27	4.3^a^ ± 0.23	6.6^c^ ± 0.81	6.4^c^ ± 0.07	6.5^c^ ± 0.12	5.5^b^ ± 0.33	6.1^c^ ± 0.07	4.75
	(7.6)	(3.6)	(5.9)	(5.6)	(5.8)	(4.6)	(5.1)	
L/C_A_	0.11^e^ ± 0.003	0.07^a^ ± 0.004	0.10^d^ ± 0.013	0.09^c^ ± 0.001	0.09^c^ ± 0.001	0.08^b^ ± 0.004	0.08^bc^ ± 0.001	5.44
Method B								
Glucan	36.2 ± 0.55	39.8 ± 0.88	41.8 ± 1.53	34.4 ± 1.89	36.9 ± 3.01	39.5 ± 0.36	37.9 ± 2.01	3.48
	(32.6)	(33.6)	(37.4)	(30.0)	(32.5)	(33.2)	(32.5)	
GAX	23.9 ± 0.36	20.1 ± 0.65	22.9 ± 1.29	23.6 ± 0.30	24.9 ± 0.83	22.4 ± 0.38	21.9 ± 0.72	4.27
	(21.6)	(17.0)	(20.5)	(20.6)	(21.9)	(18.9)	(18.7)	
Py.lignin^1^	24.0 ± 0.83	8.6 ± 0.36	9.6 ± 0.72	14.2 ± 0.72	16.1 ± 1.07	10.7 ± 0.39	10.9 ± 0.29	4.76
	(21.6)	(7.3)	(8.6)	(12.4)	(14.1)	(8.9)	(9.2)	
L/C_B_	0.52 ± 0.022	0.15 ± 0.008	0.15 ± 0.017	0.28 ± 0.019	0.26 ± 0.017	0.18 ± 0.020	0.21 ± 0.006	5.34

Values with different superscripts within a row are significantly (*P* < 0.05) different. No analysis of variance (ANOVA) was carried out on Method B data and the values are mean of analytical replicates; CV, coefficient of variation; IVGP, total *in vitro* gas production after 72 h incubation in the rumen; OM, organic matter; DM, dry matter; CP, crude protein (N × 6.25); Method A, Van Soest *et al.* 8; Cell, cellulose; Hcell, hemicellulose; ADL, acid‐detergent lignin; L/C_A_, ratio of ADL/(Cell + Hcell); Method B, Py‐GC/MS and monosaccharide analysis for lignin and carbohydrates, respectively; GAX, glucuronoarabinoxylan; L/C_B_, ratio of lignin/(Glucan + GAX).

1 Lignin content as estimated using carbon 13 (^13^C) quantitative Py‐GC/MS.

In Method B, the cellulose content (measured as released glucose after hydrolysis) was 15.8% to 32.2% lower, as compared to cellulose measured with Method A. It is inferred that Method A may overestimate the cellulose content since parts of hemicellulose (e.g. xylan that is associated with lignin) can also end up in the ADF fraction (Fig. 1). In addition, a significant portion of lignin is solubilized by the acid‐detergent solution, leading to overestimation of cellulose contents.^24^ Similarly, the limitations of ADF (and NDF) lead to inaccuracies in the quantification of hemicellulose. In Method B, hemicellulose content (measured as GAX) of untreated straw was 17.8% lower than hemicellulose estimated by the Method A. One of the possible reasons is that not all hemicellulose in the untreated straw can be hydrolyzed in Method B due to interaction with other cell wall components. Upon fungal pretreatment, partly degraded hemicellulose may be solubilized in Method A, but can be measured as the constituent monosaccharides in Method B. All fungal‐treated wheat straw showed 1.5% to 28.5% higher GAX contents, as compared to the hemicellulose obtained by Method A. The lignin contents as determined using ^13^C quantitative Py‐GC/MS (Py.lignin; Method B) were 1.5 to 2.8 times higher, as compared to ADL in Method A. This difference is expected since ADL greatly underestimates the total lignin contents.^9^ Between the two methods, good correlations were only found in the determination of hemicellulose–GAX (*r* = 0.76; *P* < 0.001) and ADL–Py.lignin (*r* = 0.84; *P* < 0.001).

### Mass recovery and the degradation of cell wall components

To demonstrate the disparities in mass recoveries by both methods (Fig. 1), the degradation of different cell wall components (in absolute amounts) were assessed. For different fungal‐treated samples, both methods only resulted in a similar assessment of hemicellulose and lignin degradation. This was not the case for cellulose, where both methods resulted in a slight difference in the assessment of cellulose degradation for some samples. For example, both *C. subvermispora* strains degraded ∼4% of cellulose based on Method A. In contrast, based on Method B, both strains apparently increased the amount of cellulose by ∼9%.

The amount of cellulose should not increase, since this polysaccharide is used by fungi for growth (mass transfer) and metabolism. The monosaccharides analysis^13^ can also include the non‐structural carbohydrates, which are soluble in neutral‐ and acid‐detergent solutions. Similar to hemicellulose, a more complete hydrolysis of cellulose upon fungal pretreatment can be achieved in Method B, leading to an apparent increase of the cellulose content. In the untreated straw, cellulose crystallinity and interaction with other cell wall components may limit a complete hydrolysis. We also speculate an interference from the fungal biomass with the cellulose and glucan contents (Fig. 1). True (higher) fungi do not contain cellulose, a *β*‐(1→4)‐d‐glucan polymer.^25^ However, fungal polysaccharides such as *β*‐(1→3)‐d‐glucan and *α*‐(1→3)‐d‐glucan 26, as well as chitin (*β*‐(1→4)‐linked *N*‐acetyl‐d‐glucosamine), may be included in the total cellulose content of Method A. Meanwhile, the acid hydrolysis step in Method B will also hydrolyze all glucan belonging to the fungal biomass; but not chitin, which requires a stronger acid.^27^ Due to its recalcitrant nature, chitin may end up in the ADL fraction. Hence, it can be concluded that none of the two methods allow accurate quantification of straw cellulose without interference from fungal glucan.

Comparing the two methods, the mean degradation of GAX for different samples was only 9.3%, as compared to hemicellulose in Method A (33.4%). Py.lignin gave a higher degradation of lignin (61.9%), compared to the ADL (33.2%). These large differences between methods indicate disparities in the mass recovery of cell wall components, which may change the assessment made on the fungal modification of lignocellulosic biomass. Using Method A, it has been reported that the degradation of ADL is accompanied by the degradation of hemicellulose.^28^ In the present study, the degradation of hemicellulose (20.0% to 49.7%) followed a similar pattern to that of ADL (22.3% to 52.2%). A significant (*P* < 0.001) correlation of ADL with hemicellulose was observed (*r* = 0.93) (Table 2). Method B, however, suggests a lesser degradation of hemicellulose (determined as GAX), i.e. both polymers are not simultaneously degraded by fungi (at a comparable magnitude), as commonly reported in the literature.^4^, ^7^, ^15^ The fragmented lignin may also contain intact hemicellulose polymers, especially xylan,^29^ which explains the strong correlation in Method A (simultaneous ‘degradation’ of ADL and hemicellulose).

**Table 2:** Lignin sub‐units (mean ± SD) in wheat straw treated with different fungal strains for 7 weeks, using carbon 13 (^13^C) quantitative Py‐GC/MS

		*Ceriporiopsis subvermispora* strain		*Pleurotus eryngii* strain		*Lentinula edodes* strain	
Parameter	Control	1	12	3	6	8	10
Lignin sub‐units (%)							
H	9.6 ± 0.40	12.0 ± 0.74	12.1 ± 0.26	10.0 ± 0.71	10.1 ± 0.79	11.1 ± 0.47	11.3 ± 0.46
G	62.2 ± 0.66	62.1 ± 0.52	59.3 ± 0.29	62.8 ± 1.40	63.3 ± 0.27	64.2 ± 0.24	63.0 ± 0.56
S	28.2 ± 0.54	26.0 ± 0.42	28.6 ± 0.07	27.2 ± 0.78	26.7 ± 0.93	24.7 ± 0.37	25.6 ± 0.13
S/G ratio	0.45 ± 0.01	0.42 ± 0.01	0.48 ± 0.00	0.43 ± 0.02	0.42 ± 0.02	0.38 ± 0.01	0.41 ± 0.01

No analysis of variance (ANOVA) was carried out on the data and the values are mean of analytical replicates. A complete characterization of lignin structural moieties (e.g. unsubstituted and C_*α*_‐oxidized compounds) was provided in Van Erven *et al.* 12

H, *p*‐hydroxyphenyl unit; G, guaiacyl lignin subunit; S, syringyl unit.

**Figure 2:**
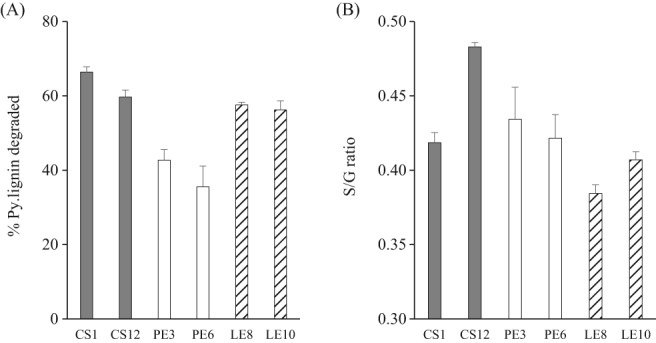
(A) Degradation (%) of Py.lignin as quantified using Py‐GC/MS with ^13^C as an internal standard for 7 weeks of treatment of wheat straw with *Ceriporiopsis subvermispora* (CS1, CS12), *Pleurotus eryngii* (PE3, PE6) and *Lentinula edodes* (LE8, LE10). (B) The ratio of syringyl (S) to guaiacyl (G) ratio for each treated straw. Error bars indicate standard deviation.

Lignin is the main determinant of ruminal digestiblity.^24^, ^30^ Therefore, an accurate and in‐depth assessment of the lignin content and structure is crucial, which can be obtained by using an advanced analysis such as Py‐GC/MS.^10^, ^11^ The ^13^C quantitative Py‐GC/MS of the samples released 49 lignin‐derived compounds (see Table S1), which were used to quantify the Py.lignin contents (Table 1). Based on this method, a species‐dependent trend in the extent of Py.lignin degradation can be observed, with *C. subvermispora* strains degrading most lignin (∼63%), followed by *L. edodes* strains (∼58%) and *P. eryngii* (∼39%) (Fig. 2). These figures were remarkably higher, as compared to the assessment using Method A, where *C. subvermispora*, *L. edodes* and *P. eryngii* strains degraded ∼40%, 36% and 26% of ADL, respectively. No clear‐cut species‐dependent trend can be observed from Method A. Both methods provided a notable difference in the degradation of lignin for a *C. subvermispora* strain, CS12 (22.3% *versus* 69.7% for Method A and B, respectively). It was however unclear on the lower lignin degradation observed for CS12‐treated wheat straw, using Method A.

Besides content, Py‐GC/MS also provides information on the lignin structural features (Table 2), which is a substantial advantage over Method A, particularly in providing insight on the underlying mechanism of lignin degradation. By referring to a lower lignin degradation observed for CS12‐treated straw in Method A – indeed, CS12 showed a slight preference towards degrading guaiacyl (G) unit compounds, as compared to other fungi (Fig. 2). However, the overall results showed that the preference of these high potential fungi was almost non‐existent, indicating the ability of the majority of these fungi to degrade different monolignol units at the same magnitude. In the literature, however, most fungi showed a preference in degrading the syringyl (S) unit compounds, compared to the G‐units.^5^, ^31^, ^32^ The S‐unit compounds have a slightly lower redox potential and fewer aryl‐*O*‐aryl bonds, making them more susceptible to fungal degradation.^31^ We have recently provided a detailed insight into the mechanisms of these selected fungi in degrading and modifying different lignin moieties, through a combination of Py‐GC/MS and nuclear magnetic resonance (NMR) analyses.^12^

### Relationship of cell wall contents with ruminal digestibility

To facilitate further understanding, we compared the two methods based on the relationship of cell wall contents with ruminal digestibility, as measured by the IVGP.^14^ The changes in IVGP of these fungal‐treated straws have been previously discussed in detail^16^ – hence, will only be mentioned briefly. All fungi clearly increased the IVGP of the wheat straw (Table 1). Still, large IVGP variations were observed among different species (to a lesser extent, between strains of the same species), with ∼24% difference between CS1 (highest) and PE3 (lowest). A species‐dependent trend, similar to the degradation of Py.lignin (Method B) was also observed. Data at different time points (weeks 1, 3 and 7) were used for comparing the correlations among all measured variables from the two methods (Table 3). Previously, we showed that most changes (cell wall contents and IVGP) were observed at these time points.^4^ Control (week 0) data were excluded from the correlation analysis. The weekly data for Py.lignin contents (Method B) were based on semi‐quantitative Py‐GC/MS, which showed ∼9% higher Py.lignin contents, as compared to the ^13^C quantitative Py‐GC/MS.

**Table 3:** Pearson's correlations coefficient (r) among the cell wall contents (% w/w dry matter), measured using different methods, to the in vitro gas production (IVGP) of the wheat straw treated with different fungal strains for 1, 3 and 7 weeks

	Method A					Method B			
	IVGP	Cell	Hcell	ADL		IVGP	Glu	GAX	Py.lignin
IVGP	1				IVGP	1			
Cell	0.46	1			Glu	0.39	1		
Hcell	−0.84***	−0.62**	1		GAX	−0.53*	−0.53*	1	
ADL	−0.88***	−0.62**	0.93***	1	Py.lignin	−0.73**	−0.65**	0.61*	1

IVGP, total *in vitro* gas production after 72 h incubation in the rumen; Method A, Van Soest *et al.* 8; Cell, cellulose; Hcell, hemicellulose; ADL, acid‐detergent lignin; Method B, semi‐quantitative Py‐GC/MS and monosaccharide analysis for Py.lignin and carbohydrates, respectively; Glu, glucan; GAX, hemicellulosic sugar (glucuronoarabinoxylan).

Significance of the correlation coefficient (*r*):

* *P* < 0.05;

** *P* < 0.01;

*** *P* < 0.001.

**Figure 3:**
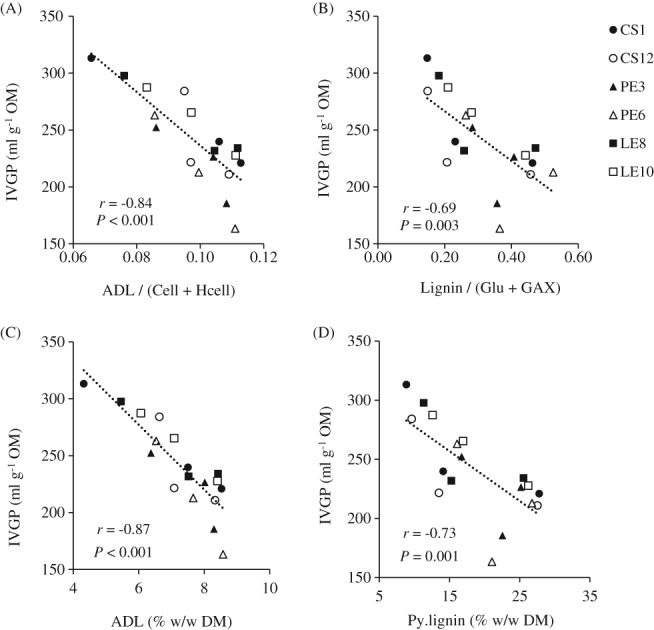
Correlations plot for the relationships between *in vitro* gas production (IVGP), lignin to total structural carbohydrates (L/C) ratio and lignin, determined by using Method A (A, C) and Method B (B, D). Method A is based on Van Soest method^8^ and Method B is based on semi‐quantitative Py‐GC/MS^11^ and monosaccharide analyses.^13^ Data from weeks 1, 3 and 7 were used. The untreated wheat straw (control; week 0) was excluded from the correlation analysis. Each point indicates average of three biological or technical replicates for Methods A and B, respectively. ADL, acid‐detergent lignin; Cell, cellulose; Hcell, hemicellulose; Glu, glucan; GAX, glucuronoarabinoxylan. Respective Pearson's *r* and *P* values are indicated.

With Method A, the IVGP was positively correlated to cellulose (*r* = 0.46; *P* = 0.070), but negatively correlated to hemicellulose (*r* = −0.84; *P* < 0.001) and ADL (*r* = −0.88; *P* < 0.001) (Table 3). These results are consistent with previous reports, which used the same Method A.^4^, ^15^ The high correlation of ADL with the IVGP (Fig. 3) is expected since the ADL fraction only accounts for the recalcitrant lignin, which is highly resistant to fermentation in the rumen. This recalcitrant lignin covalently bonds to its associated polysaccharides, which leads to a low degradability of cell walls in the rumen.^30^ It is inferred that the residual lignin in the ADL fraction may be structurally modified, but is not completely degraded by the fungi. Similar circumstances are also applicable to the cellulose contents, where degraded cellulose is solubilized, leaving behind intact cellulose polymers. This explains a low correlation of cellulose with the IVGP. Figure 3 shows the relationship between IVGP and the lignin to structural carbohydrates (L/C) ratio. The L/C from the two methods showed significant (*P* < 0.01) relationships with the IVGP, although Method A showed a higher correlation coefficient (*r*), compared to Method B (−0.84 *versus* −0.69, respectively). Both methods are considered reliable in explaining the subsequent IVGP of fungal‐treated wheat straw. Although both methods show the same trend, the correlation coefficients of the individual cell wall components with the IVGP were noticeably lower for Method B.

Combined with our earlier assessment on the mass recoveries, these observations led to several inferences: (i) Method A is more indicative of the cell wall solubility – which explained the IVGP well, rather than indicating the actual cell wall degradation by fungi. The more specific Method B elucidates more on the structure, which aids our understanding of variations among fungal strains in modifying the cell wall; (ii) Although the recalcitrant lignin (ADL) caused a strong negative effect on the IVGP, the inclusion of all lignin‐derived compounds in Py.lignin contents (acid soluble fraction and/or modified lignin fragments) suggested a weaker relationship. The inverse relationship of lignin with digestibility is widely accepted.^30^ In their review, Susmel and Stefanon^33^ combined results from several studies, and concluded that an ‘apparent’ digestion of lignin in the rumen (2% to 53%) occurs. However, it is still debatable if modified lignin fragments could be fermented in the rumen and contribute to the IVGP. (iii) The close association of lignin to hemicellulose described earlier, is also a valid explanation for a weaker negative relationship of Py.lignin with IVGP, as compared to ADL.

Besides interfering in the cell wall compositional analyses, the fungal glucan, like the straw cellulose, are fermentable in the rumen.^34^ Therefore, we speculate a contribution of pure mycelia in the wheat straw to the total IVGP. Although chitin is thought to be resistant to microbial degradation, several studies reported the presence of chitinolytic microbes and proved the presence of chitinase and *N*‐acetyl‐*β*‐glucosaminidase activities in the rumen.^34^, ^35^ Due to the fact that the IVGP is the cumulative production of carbon dioxide (CO_2_) (∼65%), methane (∼31%) and other minor gases^36^ from any fermentable OM, we performed a pilot trial on the fermentability of the pure fungal biomass in rumen fluid (see Supporting Information Procedure S1). Results showed that fungal biomass (based on ergosterol measurement^37^) only accounted for less than 3% of total OM of the treated wheat straw (see Table S2). The pure mycelial mass resulted in a higher IVGP compared to their respective treated straws, by a factor of 1.03 to 1.20 (see Fig. S1). These results show that the fungal biomass is fermentable in the rumen and contribute to the total IVGP, although the fermentation of straw polymers is still the major part of the observed IVGP. However, the *in situ* infiltration of fungal biomass in lignocellulose matrix, may affect its effective degradability in the rumen.

Previously, Van Kuijk *et al.* 5 showed a strong correlation (*r* = −0.75) of the S/G ratio with the IVGP of wheat straw treated with a single fungus – *L. edodes*. In the present study, there was a poor correlation of IVGP and the pooled S/G ratio (*r* = −0.28) and even to the Py.lignin contents (*r* = −0.08). Similar observations were observed when correlating IVGP with other specific structural features of lignin (data not shown). This is expected since fungi are unique in attacking certain moieties and structural features of lignin.^12^ The IVGP showed a significant correlation with the Py.lignin contents (*r* = −0.73; *P* < 0.01). These observations show that lignin content, rather than its structural features, is the main determinant for the subsequent ruminal digestibility.

### Reflection on the application of both methods for bioprocessed biomass

From ruminant perspective, ruminal digestibility – assessed either by *in vitro*, *in situ* or *in vivo* technique, is the most effective technique to select the best fungi for improving the nutritive value of wheat straw.^16^ To explain the changes in the digestibility, Method A should be used. This method is preferred by many ruminant nutritionists as it is relatively easy to perform with high‐throughput. However, in assessing fungal‐treated biomass, the accuracy of Method A greatly relies on the precision of the extraction and filtration steps.^38^ Smaller lignin and structural carbohydrates degradation products can easily be removed from the normal pore size filter bags (*Φ* = 25 µm). Therefore, Method A should not be used as the sole method to conclude on the capability of a particular fungus in degrading different components of cell wall. For a fundamental understanding of fungal modification of cell wall, Method B (Py‐GC/MS in particular) should be used instead. While lignin can be accurately quantified by Py‐GC/MS, neither methods assessed here can provide a satisfactory assessment of the cellulose – among others, due to the interference from fungal biomass. Therefore, a more advanced technique such as radioactive labelling (e.g. ^13^C) of substrate (or fungi)^39^ should be considered to unravel the complex cell wall modification by white‐rot fungi.

## CONCLUSION

The two methods – Method A, the Van Soest method and Method B, the constituent monosaccharide analysis and Py‐GC/MS – differ considerably in the mass recoveries of different cell wall components. Method A can be used to explain the subsequent digestibility of fungal‐treated wheat straw in the rumen, since it only accounts for the recalcitrant and insoluble residues. Method A underestimates the cell wall contents, hence, should not be solely used in assessing the capability of fungi to modify the cell wall. While Method A is relatively easy to perform with high‐throughput, Method B should be used when characterizing the underlying mechanisms of cell wall breakdown by fungi. The latter provides a more accurate quantification of cell wall, particularly lignin. Neither methods could accurately quantify the cellulose contents, which has been attributed to method limitations and the interference of fungal biomass.

## DECLARATION OF INTEREST STATEMENT

All authors have no conflict of interest to declare.

## Supporting information


**Procedure S1.** Estimation of fungal biomass and its ruminal digestibility.
**Figure S1.** The *in vitro* gas production (IVGP) of pure fungal biomass (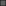) and the corresponding wheat straw (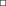) treated with *Ceriporiopsis subvermispora* (CS12), *Pleurotus eryngii* (PE6) and *Lentinula edodes* (LE8) for 7 weeks. Error bars indicate standard deviation. Bars with different superscript letters are significantly (*P* < 0.05) different.
**Table S1.** Identities and relative abundances of lignin‐derived compounds released upon 13C‐IS Py‐GC/MS of wheat straw treated with different fungal strains for 7 weeks
**Table S2.** Estimation of fungal biomass present in wheat straw treated with *Ceriporiopsis subvermispora* (CS12), *Pleurotus eryngii* (PE6) and *Lentinula edodes* (LE8) for 7 weeksClick here for additional data file.

## References

[1] Van Kuijk SJA , Sonnenberg ASM , Baars JJP , Hendriks WH and Cone JW , The effect of adding urea, manganese and linoleic acid to wheat straw and wood chips on lignin degradation by fungi and subsequent *in vitro* rumen degradation. Anim Feed Sci Technol 213:22–28 (2016).

[2] Hendriks ATWM and Zeeman G , Pretreatments to enhance the digestibility of lignocellulosic biomass. Bioresour Technol 100:10–18 (2009). 18599291 10.1016/j.biortech.2008.05.027

[3] Rabemanolontsoa H and Saka S , Various pretreatments of lignocellulosics. Bioresour Technol 199:83–91 (2016). 26316403 10.1016/j.biortech.2015.08.029

[4] Nayan N , Sonnenberg ASM , Hendriks WH and Cone JW , Differences between two strains of *Ceriporiopsis subvermispora* on improving the nutritive value of wheat straw for ruminants. J Appl Microbiol 123:352–361 (2017). 28517113 10.1111/jam.13494

[5] Van Kuijk SJA , Del Río JC , Rencoret J , Gutiérrez A , Sonnenberg ASM , Baars JJP *et al.*, Selective ligninolysis of wheat straw and wood chips by the white‐rot fungus *Lentinula edodes* and its influence on *in vitro* rumen degradability. J Anim Sci Biotechnol 7:1–14 (2016). 27688879 10.1186/s40104-016-0110-z PMC5034620

[6] Van Kuijk SJA , Sonnenberg ASM , Baars JJP , Hendriks WH , del Río JC , Rencoret J *et al.*, Chemical changes and increased degradability of wheat straw and oak wood chips treated with the white rot fungi *Ceriporiopsis subvermispora* and *Lentinula edodes* . Biomass Bioenergy 105:381–391 (2017).

[7] Tuyen VD , Cone JW , Baars JJ , Sonnenberg ASM and Hendriks WH , Fungal strain and incubation period affect chemical composition and nutrient availability of wheat straw for rumen fermentation. Bioresour Technol 111:336–342 (2012). 22377477 10.1016/j.biortech.2012.02.001

[8] Van Soest PJ , Robertson JB and Lewis BA , Methods for dietary fiber, neutral detergent fiber, and nonstarch polysaccharides in relation to animal nutrition. J Dairy Sci 74:3583–3597 (1991). 1660498 10.3168/jds.S0022-0302(91)78551-2

[9] Jung HJG , Varel VH , Weimer PJ and Ralph J , Accuracy of Klason lignin and acid detergent lignin methods as assessed by bomb calorimetry. J Agric Food Chem 47:2005–2008 (1999). 10552486 10.1021/jf981250q

[10] Del Río JC , Rencoret J , Prinsen P , Martínez ÁT , Ralph J and Gutiérrez A , Structural characterization of wheat straw lignin as revealed by analytical pyrolysis, 2D‐NMR, and reductive cleavage methods. J Agric Food Chem 60:5922–5935 (2012). 22607527 10.1021/jf301002n

[11] Van Erven G , De Visser R , Merkx DWH , Strolenberg W , De Gijsel P , Gruppen H *et al.*, Quantification of lignin and its structural features in plant biomass using ^13^C lignin as internal standard for pyrolysis‐GC‐SIM‐MS. Anal Chem 89:10907–10916 (2017). 28926698 10.1021/acs.analchem.7b02632 PMC5647568

[12] Van Erven G , Nayan N , Sonnenberg ASM , Hendriks WH , Cone JW and Kabel MA , Mechanistic insight in the selective delignification of wheat straw by three white‐rot fungal species through quantitative ^13^C‐IS py‐GC–MS and whole cell wall HSQC NMR. Biotechnol Biofuels 11:1–16 (2018). 30263063 10.1186/s13068-018-1259-9 PMC6156916

[13] Englyst HN and Cummings JH , Simplified method for the measurement of total non‐starch polysaccharides by gas‐liquid chromatography of constituent sugars as alditol acetates. Analyst 109:937 (1984). 10.1039/an9820700307 6283946

[14] Cone JW , van Gelder AH , Visscher GJW and Oudshoorn L , Influence of rumen fluid and substrate concentration on fermentation kinetics measured with a fully automated time related gas production apparatus. Anim Feed Sci Technol 61:113–128 (1996).

[15] Van Kuijk SJA , Sonnenberg ASM , Baars JJP , Hendriks WH and Cone JW , Fungal treatment of lignocellulosic biomass: importance of fungal species, colonization and time on chemical composition and *in vitro* rumen degradability. Anim Feed Sci Technol 209:40–50 (2015).

[16] Nayan N , Sonnenberg ASM , Hendriks WH and Cone JW , Screening of white‐rot fungi for bioprocessing of wheat straw into ruminant feed. J Appl Microbiol 125:468–479 (2018). 29704882 10.1111/jam.13894

[17] Jurak E , Punt AM , Arts W , Kabel MA and Gruppen H , Fate of carbohydrates and lignin during composting and mycelium growth of *Agaricus Bisporus* on wheat straw based compost. PLoS One 10:1–16 (2015). 10.1371/journal.pone.0138909 PMC4593547 26436656

[18] Thibault JF , Automatisation du dosage des substances pectiques par la methode au meta‐hydroxydiphenyl. Leb Wiss Technol 12:247–251 (1979).

[19] Ralph J and Hatfield RD , Pyrolysis‐GC‐MS characterization of forage materials. J Agric Food Chem 39:1426–1437 (1991).

[20] Udén P , Robinson PH , Mateos GG and Blank R , Use of replicates in statistical analyses in papers submitted for publication in Animal Feed Science and Technology. Anim Feed Sci Technol 171:1–5 (2012).

[21] Sun XF , Sun RC , Tomkinson J and Baird MS , Degradation of wheat straw lignin and hemicellulosic polymers by a totally chlorine‐free method. Polym Degrad Stab 83:47–57 (2004).

[22] Kabel MA , Bos G , Zeevalking J , Voragen AGJ and Schols HA , Effect of pretreatment severity on xylan solubility and enzymatic breakdown of the remaining cellulose from wheat straw. Bioresour Technol 98:2034–2042 (2007). 17029957 10.1016/j.biortech.2006.08.006

[23] Szczodrak J , The enzymatic hydrolysis and fermentation of pretreated wood substrates. Biotechnol Adv 32:771–776 (1988). 10.1016/0734-9750(84)90003-x 14545694

[24] Jung H‐JG , Analysis of forage fiber and cell walls in ruminant nutrition. J Nutr 127:819S–823S (1997). 9164242 10.1093/jn/127.5.810S

[25] Badreddine I , Lafitte C , Heux L , Skandalis N , Spanou Z , Martinez Y *et al.*, Cell wall chitosaccharides are essential components and exposed patterns of the phytopathogenic oomycete *Aphanomyces euteiches* . Eukaryot Cell 7:1980–1993 (2008). 18806214 10.1128/EC.00091-08 PMC2583540

[26] Fesel PH and Zuccaro A , β‐Glucan: crucial component of the fungal cell wall and elusive MAMP in plants. Fungal Genet Biol 90:53–60 (2016). 26688467 10.1016/j.fgb.2015.12.004

[27] Einbu A and Vårum KM , Characterization of chitin and its hydrolysis to GlcNAc and GlcN. Biomacromolecules 9:1870–1875 (2008). 18540645 10.1021/bm8001123

[28] Van Kuijk SJA , Sonnenberg ASM , Baars JJP , Hendriks WH and Cone JW , Fungal treated lignocellulosic biomass as ruminant feed ingredient: a review. Biotechnol Adv 33:191–202 (2015). 25447421 10.1016/j.biotechadv.2014.10.014

[29] Buranov AU and Mazza G , Lignin in straw of herbaceous crops. Ind Crops Prod 28:237–259 (2008).

[30] Moore KJ and Jung H‐JG , Lignin and fiber digestion. J Range Manage 54:420–430 (2001).

[31] Del Río JC , Speranza M , Gutiérrez A , Martínez MJ and Martínez AT , Lignin attack during eucalypt wood decay by selected basidiomycetes: a Py‐GC/MS study. J Anal Appl Pyrolysis 64:421–431 (2002).

[32] Martínez AT , Rencoret J , Nieto L , Jiménez‐Barbero J , Gutiérrez A and Del Río JC , Selective lignin and polysaccharide removal in natural fungal decay of wood as evidenced by *in situ* structural analyses. Environ Microbiol 13:96–107 (2011). 21199251 10.1111/j.1462-2920.2010.02312.x

[33] Susmel P and Stefanon B , Aspects of lignin degradation by rumen microorganisms. J Biotechnol 30:141–148 (1993).

[34] Morgavi DP , Sakurada M , Tomita Y and Onodera R , Presence in rumen bacterial and protozoal populations of enzymes capable of degrading fungal cell walls. Microbiology 140:631–636 (1994). 8012585 10.1099/00221287-140-3-631

[35] Kopecný J , Hodrová B and Stewart CS , The isolation and characterization of a rumen chitinolytic bacterium. Lett Appl Microbiol 23:195–198 (1996). 8862026 10.1111/j.1472-765x.1996.tb00063.x

[36] Moate PJ , Clarke T , Davis LH and Laby RH , Rumen gases and bloat in grazing dairy cows. J Agric Sci 129:459–469 (1997).

[37] Niemenmaa O , Galkin S and Hatakka A , Ergosterol contents of some wood‐rotting basidiomycete fungi grown in liquid and solid culture conditions. Int Biodeterior Biodegradation 62:125–134 (2008).

[38] Marichal M d J , Trujillo AI , Cadenazzi M and Arias G , Fiber analysis: evaluation of screen printing fabric filters bags by three statistical approaches. Anim Feed Sci Technol 169:79–85 (2011).

[39] Wallander H , Ekblad A , Godbold DL , Johnson D , Bahr A , Baldrian P *et al.*, Evaluation of methods to estimate production, biomass and turnover of ectomycorrhizal mycelium in forests soils – a review. Soil Biol Biochem 57:1034–1047 (2013).

