# Supplementary material for: Evaluation of fungal degradation of wheat straw cell wall using different analytical methods from ruminant nutrition perspective
Source: J Sci Food Agric. 2019 Mar 13;99(8):4054–62. doi: 10.1002/jsfa.9634 (PMC6593870; doi:10.1002/jsfa.9634)
Supplement: Supplementary file 1 — Procedure S1. Estimation of fungal biomass and its ruminal digestibility. Figure S1. The in vitro gas production (IVGP) of pure fungal biomass () and the corresponding wheat straw () treated with Ceriporiopsis subvermispora (CS12), Pleurotus eryngii (PE6) and Lentinula edodes (LE8) for 7 weeks. Error bars indicate standard deviation. Bars with different superscript letters are significantly (P < 0.05) different. Table S1. Identities and relative abundances of lignin‐derived compounds released upon 13C‐IS Py‐GC/MS of wheat straw treated with different fungal strains for 7 weeks Table S2. Estimation of fungal biomass present in wheat straw treated with Ceriporiopsis subvermispora (CS12), Pleurotus eryngii (PE6) and Lentinula edodes (LE8) for 7 weeks [file JSFA-99-4054-s001.docx]

Supporting information for:

**Evaluation of fungal degradation of wheat straw cell wall using different analytical methods from ruminant nutrition perspective**

Nazri Nayan^a*^, Gijs van Erven^b^, Mirjam A. Kabel^b^, Anton S.M. Sonnenberg^c^, Wouter H. Hendriks^a^ and John W. Cone^a^

^a^Animal Nutrition Group, Wageningen University & Research, De Elst 1, 6708 WD, Wageningen, The Netherlands

^b^Laboratory of Food Chemistry, Wageningen University & Research, Bornse Weilanden 9, 6708 WG, Wageningen, The Netherlands

^c^Plant Breeding, Wageningen University & Research, Droevendaalsesteeg 1, 6708 PB, Wageningen, The Netherlands

*Correspondence: nazri.nayan@live.com. Animal Nutrition Group, Wageningen University & Research. P.O. Box 338, 6700 AH, Wageningen, the Netherlands

| **Table S1.**Identities and relative abundances of lignin-derived compounds released upon ^13^C-IS Py-GC/MS of wheat straw treated with different fungal strains for 7 weeks. | | | | | | | | | | | | | | |
| --- | --- | --- | --- | --- | --- | --- | --- | --- | --- | --- | --- | --- | --- | --- |
| Label | Compound | Origin | Ret. Time | MW ^12^C | Structure* | Control | *C. subvermispora* strain | |  | *P. eryngii*  strain | |  | *L. edodes*  strain | |
|  |  |  |  |  |  |  | 1 | 12 |  | 3 | 6 |  | 8 | 10 |
|  |  |  |  |  |  |  |  |  |  |  |  |  |  |  |
| 1 | phenol | H | 10.03 | 94 | Unsub | 0.7 | 2.0 | 1.9 |  | 1.1 | 0.9 |  | 1.5 | 1.6 |
| 2 | guaiacol | G | 10.31 | 124 | Unsub | 2.1 | 4.2 | 4.0 |  | 2.7 | 2.5 |  | 3.2 | 3.4 |
| 3 | 2-methylphenol | H | 11.29 | 108 | Methyl | 0.2 | 0.4 | 0.4 |  | 0.2 | 0.2 |  | 0.3 | 0.3 |
| 4 | 4-methylphenol | H | 12.23 | 108 | Methyl | 0.5 | 1.1 | 1.2 |  | 0.8 | 0.7 |  | 0.9 | 1.0 |
| 5 | 4-methylguaiacol | G | 13.01 | 138 | Methyl | 0.7 | 1.0 | 1.0 |  | 0.9 | 0.9 |  | 0.9 | 0.9 |
| 6 | 2,4-dimethylphenol | H | 13.46 | 122 | Methyl | 0.1 | 0.2 | 0.2 |  | 0.1 | 0.1 |  | 0.1 | 0.2 |
| 7 | 4-ethylphenol | H | 14.52 | 122 | Ethyl | 0.1 | 0.1 | 0.1 |  | 0.1 | 0.1 |  | 0.1 | 0.1 |
| 8 | 4-ethylguaiacol | G | 15.19 | 152 | Ethyl | 0.1 | 0.2 | 0.2 |  | 0.2 | 0.2 |  | 0.2 | 0.2 |
| 9 | 4-vinylguaiacol | G/FA | 16.64 | 150 | Vinyl | 20.3 | 20.4 | 20.3 |  | 19.9 | 21.4 |  | 21.7 | 21.0 |
| 10 | 4-vinylphenol | H/PCA | 16.77 | 120 | Vinyl | 7.8 | 7.7 | 7.8 |  | 7.3 | 7.8 |  | 7.8 | 7.9 |
| 11 | eugenol | G | 17.26 | 164 | Misc | 0.2 | 0.2 | 0.2 |  | 0.2 | 0.2 |  | 0.2 | 0.2 |
| 12 | 4-propylguaiacol | G | 17.34 | 166 | Misc | 0.1 | 0.2 | 0.2 |  | 0.2 | 0.2 |  | 0.2 | 0.2 |
| 13 | syringol | S | 18.00 | 154 | Unsub | 1.9 | 3.2 | 3.5 |  | 2.3 | 2.3 |  | 2.4 | 2.7 |
| 14 | *cis*-isoeugenol | G | 18.63 | 164 | Misc | 0.1 | 0.1 | 0.1 |  | 0.1 | 0.1 |  | 0.1 | 0.1 |
| 15 | 4-propenylphenol | H | 19.54 | 134 | Misc | 0.1 | 0.1 | 0.1 |  | 0.1 | 0.1 |  | 0.1 | 0.1 |
| 16 | *trans*-isoeugenol | G | 19.9 | 164 | Misc | 0.8 | 1.0 | 0.9 |  | 1.1 | 1.1 |  | 1.1 | 1.1 |
| 17 | 4-methylsyringol | S | 20.26 | 168 | Methyl | 0.5 | 0.6 | 0.6 |  | 0.6 | 0.6 |  | 0.6 | 0.6 |
| 18 | vanillin | G | 20.35 | 152 | C*_α_* -ox | 1.2 | 2.1 | 2.3 |  | 1.4 | 1.2 |  | 1.4 | 1.4 |
| 19 | 4-propyneguaiacol | G | 20.64 | 162 | Misc | 0.1 | 0.1 | 0.1 |  | 0.1 | 0.1 |  | 0.1 | 0.1 |
| 20 | 4-alleneguaiacol | G | 20.90 | 162 | Misc | 0.1 | 0.1 | 0.1 |  | 0.1 | 0.1 |  | 0.1 | 0.1 |
| 21 | homovanillin | G | 21.81 | 166 | C*_β_* -ox | 0.5 | 0.7 | 0.8 |  | 0.5 | 0.5 |  | 0.5 | 0.5 |
| 22 | 4-ethylsyringol | S | 22.00 | 182 | Ethyl | 0.0 | 0.0 | 0.1 |  | 0.1 | 0.0 |  | 0.0 | 0.0 |
| 23 | acetovanillone | G | 22.27 | 166 | C*_α_* -ox | 0.3 | 1.1 | 1.2 |  | 0.6 | 0.5 |  | 0.6 | 0.7 |
| 24 | 4-hydroxybenzaldehyde | H | 23.01 | 122 | C*_α_* -ox | 0.2 | 0.3 | 0.3 |  | 0.2 | 0.1 |  | 0.2 | 0.2 |
| 25 | 4-vinylsyringol | S | 23.32 | 180 | Vinyl | 2.4 | 2.0 | 2.2 |  | 2.3 | 2.4 |  | 2.3 | 2.3 |
| 26 | guaiacylacetone | G | 23.50 | 180 | C*_β_* -ox | 0.3 | 0.7 | 0.7 |  | 0.4 | 0.3 |  | 0.4 | 0.4 |
|  |  |  |  |  |  |  |  |  |  |  |  |  |  |  |

| **Table S1.** (*Continued*) | | | | | | | | | | | | | | |
| --- | --- | --- | --- | --- | --- | --- | --- | --- | --- | --- | --- | --- | --- | --- |
|  |  |  |  |  |  |  |  |  |  |  |  |  |  |  |
| 27 | 4-allylsyringol | S | 23.75 | 194 | Misc | 0.2 | 0.2 | 0.2 |  | 0.2 | 0.2 |  | 0.2 | 0.2 |
| 28 | propiovanillone | G | 24.18 | 180 | C*_α_* -ox | 0.0 | 0.1 | 0.1 |  | 0.0 | 0.0 |  | 0.1 | 0.1 |
| 29 | guaiacyl vinyl ketone | G | 24.47 | 178 | C*_α_* -ox | 0.1 | 0.2 | 0.2 |  | 0.1 | 0.1 |  | 0.1 | 0.1 |
| 30 | vanilloyl acetaldehyde | G | 24.69 | 194 | C*_α_* -ox,  C*_γ_* -ox | 0.2 | 4.3 | 3.9 |  | 0.9 | 0.6 |  | 1.5 | 1.5 |
| 31 | *cis*-4-propenylsyringol | S | 24.88 | 194 | Misc | 0.1 | 0.1 | 0.1 |  | 0.1 | 0.1 |  | 0.1 | 0.1 |
| 32 | 4-propynesyringol | S | 25.52 | 192 | Misc | 0.1 | 0.1 | 0.1 |  | 0.1 | 0.2 |  | 0.1 | 0.1 |
| 33 | 4-allenesyringol | S | 25.73 | 192 | Misc | 0.1 | 0.1 | 0.1 |  | 0.1 | 0.1 |  | 0.1 | 0.1 |
| 34 | *trans*-4-propenylsyringol | S | 26.19 | 194 | Misc | 0.9 | 0.8 | 0.9 |  | 1.0 | 0.6 |  | 0.9 | 0.9 |
| 35 | dihydroconiferyl alcohol | G | 26.22 | 182 | C*_γ_* -ox | 0.1 | 0.1 | 0.1 |  | 0.1 | 0.1 |  | 0.1 | 0.1 |
| 36 | syringaldehyde | S | 26.90 | 182 | C*_α_* -ox | 0.7 | 0.8 | 1.0 |  | 0.7 | 0.7 |  | 0.6 | 0.7 |
| 37 | *cis*-coniferyl alcohol | G | 26.76 | 180 | C*_γ_* -ox | 1.3 | 1.1 | 1.0 |  | 1.4 | 1.3 |  | 1.3 | 1.3 |
| 38 | homosyringaldehyde | S | 27.75 | 196 | C*_β_* -ox | 0.3 | 0.4 | 0.5 |  | 0.3 | 0.3 |  | 0.3 | 0.3 |
| 39 | acetosyringone | S | 28.20 | 196 | C*_α_* -ox | 0.6 | 1.2 | 1.4 |  | 0.9 | 0.8 |  | 0.8 | 0.8 |
| 40 | *trans*-coniferyl alcohol | G | 28.55 | 180 | C*_γ_* -ox | 31.6 | 22.4 | 20.0 |  | 30.0 | 29.8 |  | 28.3 | 27.7 |
| 41 | *trans*-coniferaldehyde | G | 28.92 | 178 | C*_γ_* -ox | 1.8 | 2.0 | 1.8 |  | 2.1 | 1.9 |  | 2.0 | 1.9 |
| 42 | syringylacetone | S | 29.13 | 210 | C*_β_* -ox | 0.3 | 0.7 | 0.8 |  | 0.4 | 0.4 |  | 0.5 | 0.5 |
| 43 | propiosyringone | S | 29.75 | 210 | C*_α_* -ox | 0.0 | 0.1 | 0.1 |  | 0.1 | 0.1 |  | 0.0 | 0.1 |
| 44 | syringoyl acetaldehyde | S | 29.90 | 224 | C*_α_* -ox,  C*_γ_* -ox | 0.2 | 3.4 | 4.0 |  | 1.0 | 0.9 |  | 1.1 | 1.4 |
| 45 | syringyl vinyl ketone | S | 30.03 | 208 | C*_α_* -ox | 0.0 | 0.1 | 0.1 |  | 0.1 | 0.1 |  | 0.0 | 0.0 |
| 46 | dihydrosinapyl alcohol | S | 30.7 | 212 | C*_γ_* -ox | 0.0 | 0.0 | 0.0 |  | 0.0 | 0.0 |  | 0.0 | 0.0 |
| 47 | *cis*-sinapyl alcohol | S | 32.22 | 210 | C*_γ_* -ox | 0.9 | 0.6 | 0.6 |  | 0.8 | 0.8 |  | 0.7 | 0.7 |
| 48 | *trans*-sinapyl alcohol | S | 33.94 | 210 | C*_γ_* -ox | 16.6 | 9.3 | 10.0 |  | 13.6 | 13.7 |  | 11.6 | 11.9 |
| 49 | *trans*-sinapaldehyde | S | 34.15 | 208 | C*_γ_* -ox | 2.0 | 2.3 | 2.4 |  | 2.4 | 2.3 |  | 2.1 | 2.0 |
|  |  |  |  |  |  |  |  |  |  |  |  |  |  |  |
| G: guaiacyl unit, H: *p*-hydroxyphenyl unit, S: syringyl unit, FA: ferulic acid, PCA: *p*-coumaric acid; Ret. Time: Retention time; MW ^12^C: Molecular weight (g/mol)  * Unsub: unsubstituted; Methyl, ethyl, vinyl: substituted compounds; Misc: miscellaneous structures; C*_α_*-ox, C*_β_*-ox, C*_γ_*-ox: compounds with oxygen at C*_α_*, C*_β_* and C*_γ_* position, respectively. More details are available in Van Erven et al. ^1^. | | | | | | | | | | | | | | |

**Procedure S1: Estimation of fungal biomass and its ruminal digestibility**

A pilot trial on the ruminal digestibility of pure fungal biomass was performed, as an attempt to show potential contribution of the biomass to the total in vitro gas production (IVGP). Only one strain per fungal species (CS12, PE6 and LE8) was randomly selected for the estimation of fungal biomass and determining the IVGP of its pure mycelial mass. These fungal strains were grown on malt extract agar plates (~300 plates per species), layered with a cellophane on the surface. After 10 days (6 days for CS12), the fully colonized mycelia were harvested. The harvested fresh biomass (3.5 to 6.9 g) was freeze-dried before being subjected to the IVGP and expressed as ml g^-1^. In the IVGP run, the corresponding fungal-treated wheat straws were also included in the same run for comparison. Fungal biomass was estimated with an ergosterol assay, which has been described in detail.^2,3^ In brief, 200 ± 10 mg sample was extracted with 10% (1:9) KOH/methanol solution, before saponification at 80°C for 60 min. Two series of extractions with distilled water and hexane followed. The collected hexane layers were pooled and dried under vacuum (Rapidvap, Kansas, MO, USA). The extracted ergosterol was re-dissolved in methanol and the solution was filtered before analysis by high performance liquid chromatography (PDA-HPLC) (Alliance HPLC system, Waters, Milford, USA). Cholecalciferol (vitamin D_3_) was used as an internal standard. The ergosterol peak was detected at 280 nm. Previous ergosterol data for wheat straw treated with the selected fungi^3^ were used in calculating the conversion factor, CF (Ergosterol _treated straw_ / Ergosterol _pure fungal biomass_) and estimating the fungal biomass (CF × total amount of organic matter, in g) present in the treated straw.

| **Table S2.** Estimation of fungal biomass present in wheat straw treated with *C. subvermispora* (CS12), *P. eryngii* (PE6) and *L. edodes* (LE8) for 7 weeks. | | | | | |
| --- | --- | --- | --- | --- | --- |
| Strain | Ergosterol (µg g^-1^) | | Conversion factor^†^ | Estimated fungal biomass in treated straw^‡^ | |
|  | Treated straw | Pure fungal biomass |  | Weight (g) | % in total OM |
|  |  |  |  |  |  |
| CS12 | 59.6^b^ | 3466.7^b^ | 0.017^b^ | 1.4^b^ | 1.7^b^ |
| PE6 | 64.9^b^ | 2821.2^c^ | 0.023^ab^ | 1.9^ab^ | 2.3^ab^ |
| LE8 | 112.3^a^ | 4049.3^a^ | 0.028^a^ | 2.3^a^ | 2.8^a^ |
|  |  |  |  |  |  |
| CV | 10.59 | 7.50 | 15.28 | 16.80 | 15.79 |
|  |  |  |  |  |  |
| Values with different superscripts within column are significantly (*P* < 0.05) different. CV: coefficient of variation.^†^ Ergosterol _treated straw_ / Ergosterol _pure fungal biomass_.  ^‡^ Conversion factor × total amount of organic matter, in g. | | | | | |

ab

b

ab

b

a

a

ab

**Fig. S1.** The *in vitro* gas production (IVGP) of pure fungal biomass (■) and the corresponding wheat straw (■) treated with *C. subvermispora* (CS12), *P. eryngii* (PE6) and *L. edodes* (LE8) for 7 weeks. Error bars indicate standard deviation. Bars with different superscript letters are significantly (*P* < 0.05) different.

**References**

1. van Erven, G., Nayan, N., Sonnenberg, A. S. M., Hendriks, W. H., Cone, J. W. & Kabel, M. A. Mechanistic insight in the selective delignification of wheat straw by three white-rot fungal species through quantitative 13C-IS py-GC–MS and whole cell wall HSQC NMR. *Biotechnol. Biofuels* **11:** 1–16 (2018).

2. Niemenmaa, O., Galkin, S. & Hatakka, A. Ergosterol contents of some wood-rotting basidiomycete fungi grown in liquid and solid culture conditions. *Int. Biodeterior. Biodegradation* **62:** 125–134 (2008).

3. Nayan, N., Sonnenberg, A. S. M., Hendriks, W. H. & Cone, J. W. Screening of white‐rot fungi for bioprocessing of wheat straw into ruminant feed. *J. Appl. Microbiol.* **125:** 468–479 (2018).
